# Spaghetti meat and woody breast myopathies in broiler chickens: similarities and differences

**DOI:** 10.3389/fphys.2024.1453322

**Published:** 2024-08-26

**Authors:** Sunoh Che, Parker Hall

**Affiliations:** ^1^ Department of Animal and Avian Sciences, University of Maryland, College Park, MD, United States; ^2^ Perdue Foods LLC, Salisbury, MD, United States

**Keywords:** broiler, transcriptomics, processing, peroxy acetic acid, heritability

## Introduction

Spaghetti meat (SM) and woody breast (WB) are two economically significant myopathies affecting the quality of broiler chicken breast meat (Barbut, 2019). Another type of breast myopathy, white striping (WS), was not included in this paper as it has become a common occurrence, observed in nearly all breast fillets from commercial broiler production systems (Che et al., 2022b). These conditions lead to reduced consumer acceptance and product depreciation, causing substantial losses in the poultry industry (Barbut, 2019). SM is characterized by the separation of muscle fiber bundles in the *Pectoralis major* muscle, resulting in a mushy and stringy texture (Baldi et al., 2018). In contrast, WB is characterized by an abnormal hardening or firmness of the breast muscle, often accompanied by pale color and occasional petechial hemorrhages (Sihvo et al., 2017). This paper aims to compare and contrast SM and WB, highlighting their similarities and differences in various aspects.

## Prevalence and economic impact

The prevalence of both myopathies is substantial, with SM ranging from 35% to 36% and severe WB ranging from 7.3% to 12%, respectively (Sihvo et al., 2017; Zampiga et al., 2018; Xing et al., 2020; Che et al., 2022b; Kang et al., 2022). However, these figures likely underestimate the true incidence, particularly for WB, due to challenges in assessment methods. While SM can be visually detected (Che et al., 2022a), the palpation-based scoring of WB lacks objectivity. The phenotypic incidence of WB in broiler chickens does not align with the microscopic assessment for necrosis and fibrosis, with the incidence can be higher when evaluated microscopically (Velleman, 2020). Moreover, the dorsal recumbent syndrome (DRS), potentially associated with WB, further highlights the underreported symptoms in live birds. DRS-affected broilers inexplicably fall onto their backs, unable to right themselves, leading to the descriptive term “turtle chicken.” Ultimately, these recumbent broilers succumb to pulmonary failure, congestion, and edema, possibly due to the pressure exerted on the cardiopulmonary system (Che et al., 2023). Developing effective strategies to mitigate these myopathies and safeguard the economic viability of the poultry industry is essential.

## Risk factors

**FIGURE 1:**
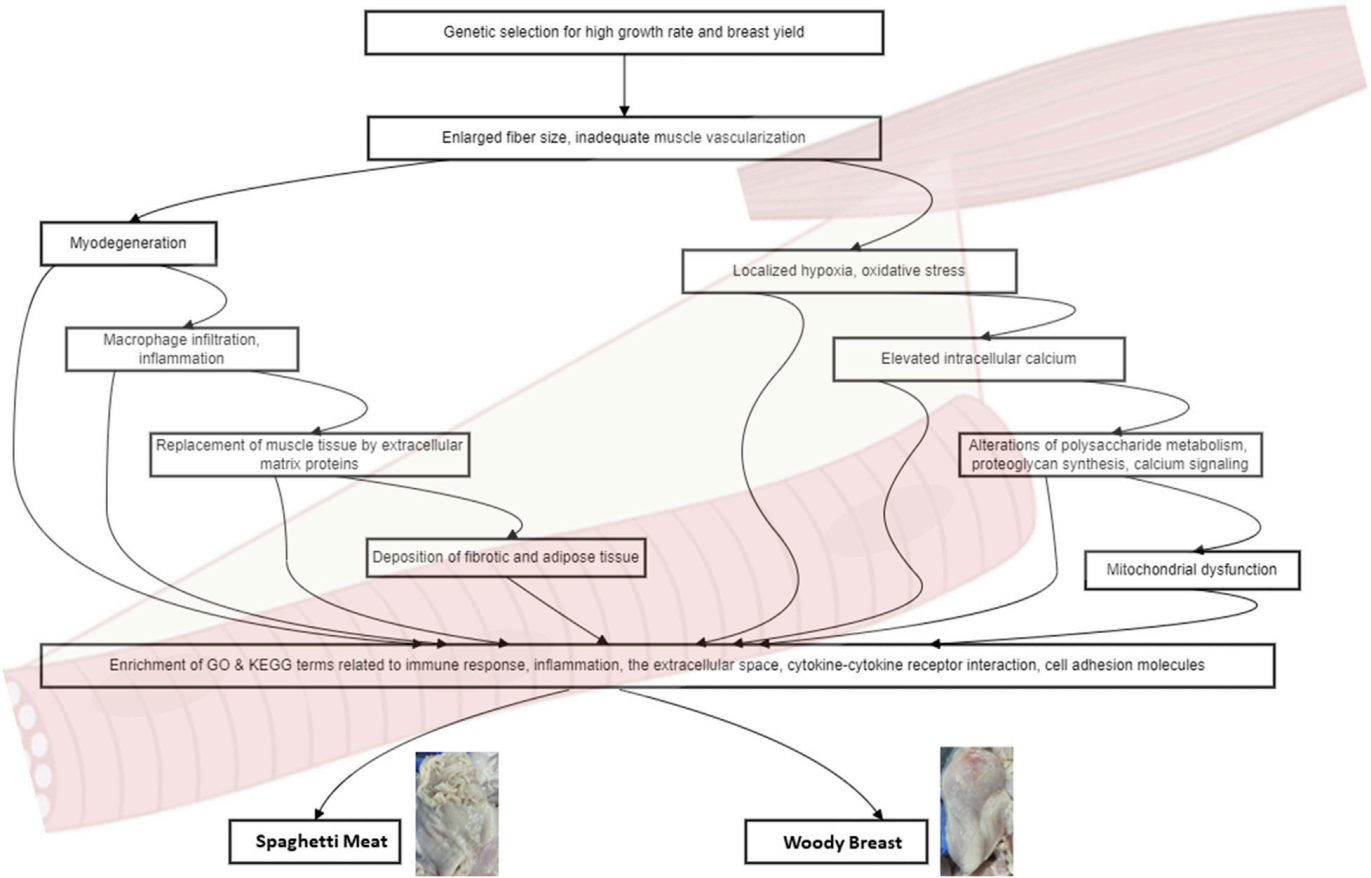
Cellular and molecular mechanisms of Woody Breast and Spaghetti Meat. The diagram shows the progression from initial genetic selection to various physiological alterations and their consequences. Genetic selection leads to enlarged fiber size and inadequate muscle vascularization, which in turn triggers two main pathways: myodegeneration and localized hypoxia/oxidative stress. The myodegeneration pathway involves macrophage infiltration, replacement of muscle tissue by extracellular matrix proteins, and deposition of fibrotic and adipose tissue. The hypoxia/oxidative stress pathway leads to elevated intracellular calcium, alterations in polysaccharide metabolism, proteoglycan synthesis, and calcium signaling, ultimately resulting in mitochondrial dysfunction. These processes converge to enrich GO and KEGG terms related to immune response, inflammation, extracellular space, cytokine-cytokine receptor interaction, and cell adhesion molecules. These molecular and cellular changes contribute to the development of two meat quality issues in poultry: Woody Breast and Spaghetti Meat.

Common risk factors, such as higher live weight at slaughter and elevated temperatures during the grow-out period, contribute to the development of both SM and WB (Che et al., 2022b). In fast-growing broiler chickens affected by WB, muscle fibers have larger diameters, and the connective tissue spacing is reduced compared to unaffected muscle (Velleman and Clark, 2015; Sihvo et al., 2018). This alteration in muscle structure leads to myodegeneration, characterized by disorganization of the sarcomeric structure (Velleman et al., 2018). In response to myodegeneration, macrophages infiltrate the affected area and secrete cytokines, promoting the replacement of muscle tissue by extracellular matrix proteins, such as collagen and proteoglycans, and the deposition of fat (Mann et al., 2011). Consequently, fibrosis occurs, characterized by an overproduction of fibrillar collagens, primarily types I and III. The organization and crosslinking of these collagens lead to the formation of collagen fibers, further contributing to the abnormal texture of the affected muscle (Velleman, 2020) (Figure 1).

Fast-growing broilers with high breast meat yields have a decreased ability to regulate their body temperature, which makes them more prone to the negative effects of thermal stress (Petracci et al., 2015). While exposure to cold temperatures can negatively impact satellite cell growth and differentiation, resulting in reduced muscle fiber size and overall muscle mass (Clark et al., 2016; Harding et al., 2016), exposure to high temperatures has been specifically associated with an increased incidence of WB and SM (Che et al., 2022b). Thermal stress, especially hot temperatures, can cause satellite cells to develop into adipose cells instead of muscle cells (Xu et al., 2022), which is associated with changes in breast meat quality, such as increased fat content (Velleman, 2023).

These findings suggest that managing growth rates and environmental conditions could be crucial in reducing the incidence of these quality defects. Interestingly, several risk factors have different effects on SM and WB. For example, coccidiosis vaccination is associated with an increased risk of SM but a decreased risk of WB. The source of chicks and hold time on lairage at the processing plant also have different effects on the two myopathies (Che et al., 2022b). These differential risk factors highlight the complexity of the underlying mechanisms and the need for targeted approaches in managing SM and WB.

### Transcriptomic profiles and molecular mechanisms

Multiple studies have revealed key molecular features and pathways associated with WB. These include localized hypoxia, oxidative stress, elevated intracellular calcium, and potential muscle fiber-type switching, which were supported by observed microscopic lesions (Mutryn et al., 2015). Alterations in pathways related to muscle development, polysaccharide metabolism, proteoglycan synthesis, inflammation, and calcium signaling have been observed (Zambonelli et al., 2016). Age-dependent transcriptional differences in WB development involve changes in glucose and lipid metabolism, cell junction dynamics, and various signaling pathways (Malila et al., 2021). Mitochondrial dysfunction in WB muscle is characterized by elevated monoacylglycerol levels, downregulation of lipid production genes, impaired fatty acid β-oxidation, and oxidative phosphorylation (Wang et al., 2023). It was suggested that selection for fast growth and breast meat yield has induced metabolic shifts towards alternative catabolic pathways for energy production, leading to oxidative stress and the initiation of inflammatory, regenerative, and fibrotic processes. (Pampouille et al., 2019). A transcriptomic meta-analysis revealed critical pathological processes in WB, including fibrosis, apoptosis, and alterations in Ca^2+^-related signaling, as well as suppression of the tricarboxylic acid cycle and mitochondrial electron transport chain (Zhang et al., 2024).

While fewer studies have focused specifically on SM, recent research has revealed important insights. Both SM and WB samples exhibit significantly elevated mRNA levels of vimentin (VIM) and desmin (DES) genes, which encode essential components of the extra-sarcomeric cytoskeleton in muscle cells, compared to normal tissue. Their analysis revealed significantly elevated mRNA levels of both *VIM* and *DES* in WB and SM samples compared to normal tissue (Soglia et al., 2020).

Comparative analysis of SM and WB transcriptomes has revealed a high degree of similarity in their transcriptomic profiles (Che et al., 2024). Compared to normal breast muscle, both SM and WB samples exhibit a substantial number of differentially expressed genes, indicating extensive transcriptomic changes associated with the development of these myopathies. Gene Ontology (GO) enrichment analysis reveals an enrichment of terms related to immune response, inflammation, and the extracellular space. Kyoto Encyclopedia of Genes and Genomes (KEGG) pathway analysis further supports the involvement of immune and inflammatory processes, with the cytokine-cytokine receptor interaction and cell adhesion molecules pathways being enriched in both myopathies (Figure 1). The lack of significant differences in gene expression between SM and WB samples suggests that these myopathies may share a common pathogenesis at the molecular level. This finding has important implications for understanding the underlying mechanisms and developing targeted interventions.

### Divergent phenotypic manifestations

Despite sharing similar molecular mechanisms and histological features, WB and SM exhibit distinct phenotypic characteristics due to differences in the extent and timing of the pathological processes involved. WB can be observed as early as week 2 after hatch (Chen et al., 2019), while SM is not typically assessed in live birds (Cahaner, 2024).

The WB phenotype is primarily attributed to the excessive deposition of fibrous tissue within the muscle, resulting from prolonged and severe fibrosis. The fibrotic process in WB is thought to be driven by sustained hypoxia and oxidative stress, leading to the activation of pro-fibrotic signaling pathways and the excessive production of extracellular matrix components, such as collagen (Soglia et al., 2017; Petracci et al., 2019).

In contrast, the SM phenotype is believed to arise from the weakening of the connective tissue structures within the muscle, potentially due to the degradation of extracellular matrix components or alterations in the organization of muscle fibers (Baldi et al., 2018). The weakened connective tissue may render the muscle more susceptible to mechanical stress during processing steps, leading to the separation of muscle fibers (Baldi et al., 2021). This vulnerability could explain why SM prevalence is significantly higher in processing plants using water chilling compared to those using air chilling (Che et al., 2022b). The increased mechanical stress from water movement during chilling may exacerbate the condition in already weakened muscle tissue.

### Heritability and genetic factors

Recent research into the genetic basis of these myopathies has revealed important insights about their heritability, highlighting the complex nature of these conditions and the potential for genetic selection as a mitigation strategy. Heritability estimates vary significantly between WB and SM, and between purebred lines and commercial crossbreds. In purebred lines, WB shows a low heritability of 0.07, while SM demonstrates a very low heritability of 0.04 (Bailey et al., 2020). However, in commercial crossbred broilers, WB exhibits a notably higher heritability of 0.49 (Lake et al., 2022). The substantial difference in heritability estimates between purebred and crossbred populations underscores the importance of studying commercially relevant populations. Multiple quantitative trait loci associated with WB have been identified, primarily clustered on chromosome 5, providing specific genetic targets for future breeding efforts (Lake et al., 2022). The higher heritability of WB in crossbreds suggests that genetics plays a more significant role in its development than previously thought, opening up greater potential for genetic selection against this myopathy.

### Processing factors and mitigation strategies

Understanding the impact of processing factors on the incidence and severity of WB and SM is crucial for developing effective mitigation strategies. Slow cooling has been reported to increase the relative incidence of SM significantly (Anton et al., 2019). Moreover, the defeathering process, including the number and configuration of defeathering machines, can also impact SM incidence and severity (Cahaner, 2024).

Peroxyacetic acid (PAA) is an oxidizing antimicrobial agent widely used in poultry processing plants in the USA to reduce levels of foodborne pathogens like *Salmonella* on raw chicken carcasses. PAA solutions up to 2000 ppm are permitted for online reprocessing of poultry in the USA (Cano et al., 2021). While PAA is effective at inactivating bacteria, there are concerns that it may negatively impact meat quality at certain concentrations and exposure times. The effects of PAA on meat quality parameters such as texture, water-holding capacity, and drip loss are not well understood. More research is needed to investigate how PAA treatment conditions influence these important quality attributes. Interestingly, SM has been reported from Italy and Canada, where PAA was not used in processing plants. To fully understand the implications of PAA use in poultry processing, additional research is necessary. Studies should focus on how PAA affects meat quality characteristics and whether it plays a role in the occurrence of SM. Such investigations will help optimize the use of PAA while maintaining product quality and safety.

## Conclusion

In conclusion, while SM and WB share common ground in their molecular underpinnings, their distinct phenotypic characteristics, heritability patterns, and sensitivity to processing factors necessitate a multifaceted approach to mitigation. Future efforts should integrate genetic selection (particularly for WB), refined management practices, and optimized processing techniques. Additionally, further research into the potential role of antimicrobial agents like PAA in meat quality and myopathy development is warranted.
